# Biologic terrorism--responding to the threat.

**DOI:** 10.3201/eid0302.970217

**Published:** 1997

**Authors:** P K Russell

**Affiliations:** Johns Hopkins University, Baltimore, Maryland, USA.


### Biologic Terrorism — Responding to the Threat

The growing awareness of the possibility that a terrorist organization might use a biologic agent in an attack on a civilian target in the United States raises important questions about our capability as a nation to respond effectively to the threat and to deal with the consequences of an attack. The article by Kaufmann et al. in this issue of *Emerging Infectious Diseases* describes three possible biologic attack scenarios and uses an economic analysis to describe the benefits of a rapid medical response and early intervention. The authors conclude that major reductions in morbidity and mortality and consequent cost savings can be achieved by early intervention. The effectiveness of postattack intervention depends on a rapid response which requires prior planning, preparation, and training. Achieving the level of preparedness implied by the assumptions stated in the article will require a major national effort. This discussion of possible bioterrorist attack scenarios adds to a growing concern about our willingness as a nation to commit the effort and resources necessary to protect our citizens.

Biologic warfare and use of biologic weapons by terrorists have only recently been discussed openly and realistically. The fall of the Soviet Union and the defeat of Iraq uncovered extensive biologic weapons programs of surprising sophistication and diversity. The threat to the nation from biologic weapons is no longer a debate issue. Now the questions are how immediate and serious is the threat and how do we respond effectively?

Protecting the armed forces against biologic weapons, although complex and difficult, is less challenging than protecting the civilian population. The armed forces are relatively small populations that can be vaccinated against the major threat agents. Aerosols containing biologic materials can be detected at a distance, and protective masks and suits are effective. Military medical personnel are trained to recognize and treat casualties, and antibiotics, antiviral drugs, and antitoxins can be stockpiled for military contingencies. The preponderance of scientific expertise for many of the threat agents is within the military medical research laboratories, although this capability is now being seriously compromised by budget cuts and personnel reductions.

The civilian population cannot be protected in the same manner as the armed forces. We must rely heavily on our intelligence and criminal investigation agencies and on international efforts to identify specific threats and deter terrorists. We must also recognize the possibility that a determined terrorist organization may not be deterred, may evade detection, and may succeed in releasing an aerosol of a virulent bacterium, virus, or toxin in a susceptible target area such as an airport or stadium. Our current capability to effectively respond to such a scenario and minimize the impact is far less than needed.

The U.S. Armed Forces and the Department of Defense have the greatest capability in biologic defense, but the responsibility for dealing with the threat of biologic weapon use by a terrorist falls on multiple federal, state, and municipal agencies and the civilian health care community. Most of the organizations are inadequately prepared to deal effectively with the problem.

The organizational aspects of dealing with an attack on our civilian population are daunting. Responsibility for recognizing an unusual outbreak of illness that may be the result of the deliberate release of a biologic warfare agent will fall on the health care community. Early recognition will be an important factor in determining the overall outcome and will depend on the level of suspicion and knowledge of the health care providers that see the initial cases. Rapid, precise, and reliable diagnosis will be the responsibility of the federal and state public health laboratory system with help from their military colleagues. Organizing and managing the care of patients and mounting the appropriate public health response will involve local health care and municipal agencies and authorities and state public health authorities. The effectiveness of coordination, support, and leadership at the federal level may make huge differences in reducing death rates and containing the possible secondary spread of a communicable disease. The Federal Emergency Management Agency has the major responsibility for planning and coordinating the consequences phase of a federal response, but the level of preparedness at all levels will ultimately determine the outcome.

If we take the biologic warfare threat seriously, a major effort will be needed to develop contingency plans and initiate coordinated and mutually supportive programs in all involved agencies. Training and education of the health care community will require a major effort involving several major professional organizations. Developing and improving diagnostic and identification capability is essential for medical care, public health, intelligence, and law enforcement agencies and should be a national priority.

The science base needed to deal with the broad spectrum of agents on the threat list, bacteria, viruses, toxins, and parasites, is widely distributed among several federal laboratories in the Department of Health and Human Services, the Department of Defense, and the Department of Energy, as well as in universities and state public health laboratories. In addition, since many of the biologic agents are not normally large public health problems or popular subjects of scientific research, critical areas have inadequate research capability and limited expert personnel. Deficiencies in our scientific knowledge and a paucity of experts will ultimately limit our capability to rapidly and precisely identify agents and respond effectively in a crisis. For example, the global molecular epidemiology of the agents at the top of the threat list is critically important for identifying the organisms accurately and differentiating local from exotic strains. Current databases are inadequate, and no organized effort is being made to fill in the gaps.

The current public discussion of the threat of biologic terrorism is an opportunity to evaluate our collective capabilities and to assess weaknesses and vulnerabilities. Raising the level of national preparedness will require leadership and action by responsible federal agencies. A thoughtful analysis of the consequences of unpreparedness provides a mandate for action.

**Philip K. Russell**
Johns Hopkins University,
Baltimore, Maryland, USA

